# Relationship between diffraction peak, network topology, and amorphous-forming ability in silicon and silica

**DOI:** 10.1038/s41598-021-00965-5

**Published:** 2021-11-12

**Authors:** Shinji Kohara, Motoki Shiga, Yohei Onodera, Hirokazu Masai, Akihiko Hirata, Motohiko Murakami, Tetsuya Morishita, Koji Kimura, Kouichi Hayashi

**Affiliations:** 1 Research Center for Advanced Measurement and Characterization, National Institute for Materials Science, 1-2-1 Sengen, Tsukuba, Ibaraki 305-0047 Japan; 2 Department of Earth Science, ETH Zürich, Clausiusstrasse 25, 8092 Zürich, Switzerland; 3 Department of Electrical, Electronic and Computer Engineering, Faculty of Engineering, Gifu University, 1-1 Yanagido, Gifu, 501-1193 Japan; 4 Center for Advanced Intelligence Project, RIKEN, 1-4-1 Nihonbashi, Chuo-ku, Tokyo, 103-0027 Japan; 5 Institute for Integrated Radiation and Nuclear Science, Kyoto University, 2-1010 Asashiro-nishi, Kumatori-cho, Sennan-gun, Osaka, 590-0494 Japan; 6 Department of Materials and Chemistry, National Institute of Advanced Industrial Science and Technology (AIST), 1–8–31 Midorigaoka, Ikeda, Osaka 563–8577 Japan; 7 Department of Materials Science, Waseda University, 3-4-1 Ohkubo, Shinjuku, Tokyo 169-8555 Japan; 8 Kagami Memorial Research Institute for Materials Science and Technology, Waseda University, 2-8-26 Nishiwaseda, Shinjuku, Tokyo 169-0051 Japan; 9 Mathematics for Advanced Materials Open Innovation Laboratory (MathAM-OIL), AIST, c/o AIMR, Tohoku University, 2-1-1 Katahira, Aoba-ku, Sendai, 980-8577 Japan; 10 Research Center for Computational Design of Advanced Functional Materials (CD-FMat), AIST, 1-1-1 Umezono, Tsukuba, Ibaraki 305-8568 Japan; 11 Department of Physical Science and Engineering, Nagoya Institute of Technology, Gokiso-cho, Showa-ku, Nagoya, 466-8555 Japan; 12 Frontier Research Institute for Materials Research, Nagoya Institute of Technology, Gokiso-cho, Showa-ku, Nagoya, 466-8555 Japan

**Keywords:** Atomic and molecular physics, Condensed-matter physics

## Abstract

The network topology in disordered materials is an important structural descriptor for understanding the nature of disorder that is usually hidden in pairwise correlations. Here, we compare the covalent network topology of liquid and solidified silicon (Si) with that of silica (SiO_2_) on the basis of the analyses of the ring size and cavity distributions and tetrahedral order. We discover that the ring size distributions in amorphous (*a*)-Si are narrower and the cavity volume ratio is smaller than those in *a*-SiO_2_, which is a signature of poor amorphous-forming ability in *a*-Si. Moreover, a significant difference is found between the liquid topology of Si and that of SiO_2_. These topological features, which are reflected in diffraction patterns, explain why silica is an amorphous former, whereas it is impossible to prepare bulk *a*-Si. We conclude that the tetrahedral corner-sharing network of AX_2_, in which A is a fourfold cation and X is a twofold anion, as indicated by the first sharp diffraction peak, is an important motif for the amorphous-forming ability that can rule out *a*-Si as an amorphous former. This concept is consistent with the fact that an elemental material cannot form a bulk amorphous phase using melt quenching technique.

## Introduction

The absence of translational periodicity and symmetry, and the rich structural complexity make it difficult to understand the order within disorder^1,2^ in disordered materials. The advent of advanced instrumentation and measurement protocols makes it feasible to use quantum beam diffraction (X-ray diffraction (XRD) and neutron diffraction (ND)) techniques to reveal the structure of disordered materials at synchrotron and/or neutron sources^3–6^.

Amorphous (*a*)-silicon (Si) and silica (SiO_2_) are the most typical and important disordered materials in both fundamental and technological research studies. In particular, SiO_2_ is a canonical amorphous-former, whereas it is possible to synthesize *a*-Si only in a thin film owing to its poor amorphous-forming ability. The short-range structural unit of these amorphous materials is a tetrahedron, SiSi_4_ in *a*-Si and SiO_4_ in *a*-SiO_2_, and the formation of their networks is governed by the corner-sharing of tetrahedra. This corner-sharing motif is within Zachariasen’s classification^7^ for the glass formation of oxide materials, but elemental amorphous materials do not follow the rule, because it is impossible to obtain bulk amorphous silicon as mentioned above.

The structures of these materials have been studied by diffraction techniques. Laaziri et al. reported high-quality X-ray diffraction data for *a*-Si with a high-real space resolution for precisely determining the coordination number^8^. The structure of *a*-SiO_2_ has been widely studied by both X-ray^9,10^ and neutron diffraction^4,11,12^. The most important feature in the diffraction data of *a*-SiO_2_ is that the first sharp diffraction peak (FSDP)^11–14^ is observed in both X-ray and neutron diffraction data, whereas the second diffraction peak, the so-called principal peak (PP)^12,14^, can be observed in only the neutron diffraction data because this peak reflects the packing of oxygen atoms^12,15^, which is sensitive to neutrons. The origin of the FSDP has been discussed for a long time. The FSDP was first discussed in 1976^16^, although it seems that the name “FSDP” was first used by Phillips in 1981^17^. The interpretation of diffraction peaks including the FSDP was attempted in the 1980s^13,17^, as discussed in details in several papers^18–22^. It is known that the FSDP of *a*-SiO_2_ is related to the formation of the random network model proposed by Zachariasen^7^, which was extended to silicate glasses by Greaves^23^ and recently revised by Mei et al., as illustrated in Ref. 21. It was demonstrated that intermediate-range ordering arises from the periodicity of boundaries between successive cages in the network formed by the connection of regular SiO_4_ tetrahedra with shared oxygen atoms at the corners associated with the formation of a ring structure and a large cavity^21,22^. The second maximum, PP, reflects the size of the local-network-forming motif, whereas the FSDP indicates the arrangement of these motifs in an intermediate range according to Zeidler and Salmon^24^. Another interpretation of the FSDP has recently been proposed by Shi and Tanaka, who discussed local tetrahedral ordering in covalent liquids and glasses^25^ and they concluded that *a*-Si has an FSDP. Moreover, an FSDP was found in metallic glasses^26^, although Price et al. implied that some diffraction peaks from amorphous alloys are not FSDPs^13^.

**Figure 1:**
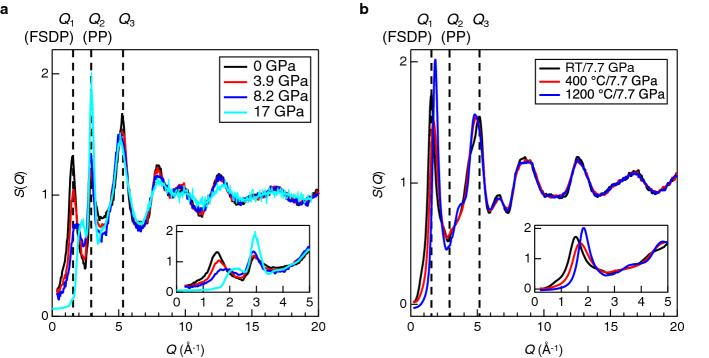
(**a**) In situ neutron structure factors, *S*(*Q*), for *a*-SiO_2_ under cold compression reported by Zeidler et al.^27^ (**b**) X-ray structure factors, *S*(*Q*), for *a*-SiO_2_ after hot compression reported by Onodera et al.^2^

The investigation of the behavior of the FSDP and PP under high pressures is important to understand the nature of intermediate-range ordering in disordered materials. Figure 1a shows in situ neutron structure factors, *S*(*Q*), for *a*-SiO_2_ under high pressures reported by Zeidler et al.^27^. It was found that the FSDP diminishes with increasing pressure, simultaneously with a peak shift to a higher *Q*. On the other hand, the PP becomes sharp with increasing pressure, suggesting that the oxygen packing fraction increases^12,15^ with the decrease of cavity volume under high pressures. This is an important benchmark for the modification of the intermediate-range ordering of *a*-SiO_2_ under cold compression. Onodera et al. recently reported the unusual behaviour of the FSDP after hot compression^2^; in the X-ray *S*(*Q*) shown in Fig. 1b, they have observed the evolution of FSDP at 7.7 GPa at a temperature higher than 400 °C. These diffraction data suggest that FSDP is very sensitive to pressure and temperature, while the PP position is insensitive to the density change.

In this article, we apply several topological techniques to analyze the ring size and cavity distributions, and tetrahedral order of crystalline, amorphous, and liquid Si and SiO_2_ to reveal the network topology^2,28–30^ for understanding the diffraction peaks in disordered materials with a special focus on the FSDP and PP.

## Methods

### Structure modeling

Atomistic models of liquid (*l*)-SiO_2_ and *a*-SiO_2_ were obtained by classical molecular dynamics (MD) simulation and MD–reverse Monte Carlo (RMC) modeling^29^, respectively. The atomistic models for *l*- and *a*-SiO_2_ were generated by combined classical MD simulation–RMC modeling. The MD model for *l*-SiO_2_ was adopted from the literature^31^. The MD simulation for *a*-SiO_2_ was performed using the large-scale atomic/molecular massively parallel simulator (LAMMPS) package^32^ within the *NVT* ensemble. The interactions were described by pair potentials with short-range Born–Mayer repulsive and long-range Coulomb terms, i.e.,

1 $${\phi }_{ij}\left(r\right)={B}_{ij}{\text{exp}}\left(-\frac{r}{{\rho }_{ij}}\right)+\frac{{e}^{2}}{4\pi {\varepsilon }_{0}}\frac{{Z}_{i}{Z}_{j}}{r},$$

where *r* is the distance between atoms *i* and *j*, *B*_*ij*_ and *ρ*_*ij*_ define the magnitude and softness of the Born–Mayer terms, respectively, *Z*_*i*_ is the effective charge on atom *i* (*Z*_Si_ = 2.4, *Z*_O_ = -1.2), *e* is the elementary charge, and *ε*_0_ is the permittivity of vacuum. The *B*_*ij*_ values were 21.39 × 10^−16^ J (Si–O), 0.6246 × 10^−16^ J (O–O) or zero (Si–Si); the *ρ*_*ij*_ values were 0.174 Å (Si–O), 0.362 Å (O–O) or zero (Si–Si). As initial configuration, 3000 (Si, 1000; O, 2000) atoms were randomly distributed in a cubic cell with a side length of 35.66 Å. The cell had a number density of 0.06615 Å^-3^. The simulation used periodic boundary conditions, and the long-range Coulomb interactions were treated by using the Ewald summation. A time step of 1 fs was used in the Verlet algorithm. The simulation temperature was maintained at 4000 K for 20,000 time steps, then the temperature was reduced to 300 K over 200,000 time steps. Finally, the system was equilibrated at 300 K for 50,000 time steps. A Nosé-Hoover thermostat was employed to control the temperature. After MD simulations, the configurations obtained for *l*- and *a*-SiO_2_ were refined by RMC modeling with constraints on the coordination numbers and on the O–Si–O bond angle distribution, in order to prevent the formation of an unfavorable disordered structure.

Atomistic models of *l*-Si and *a*-Si were obtained by RMC modeling^29^ and combined classical MD simulation–RMC modeling, respectively.

The RMC model for *l*-Si (1770 K, 5000 particles) was obtained by the RMC + + code^33^ and based on the X-ray *S*(*Q*) for *l*-Si^29^. The number density was 0.055 Å^−3^, that is consistent with a bulk mass density of 2.57 g cm^−3^^34^.

The model for *a*-Si was obtained by MD simulation, followed by RMC refinement. The MD simulation was performed using the LAMMPS code. The modified Tersoff potential ^35^ based on the three-body Tersoff potential ^36,37^ was used for describing interatomic interactions. In the modified Tersoff potential, the total energy *E* is written as follows:

2 $$E=\frac{1}{2}\sum_{i}\sum_{j\ne i}{V}_{ij}({r}_{ij}),$$

3 $${V}_{ij}\left({r}_{ij}\right)={f}_{C}\left({r}_{ij}\right)\left[A{\mathrm{exp}}\left(-{\lambda }_{1}{r}_{ij}\right)-{b}_{ij}B{\mathrm{exp}} \quad \left(-{\lambda }_{2}{r}_{ij}\right)\right],$$

4 $${f}_{C}\left(r\right)=\left\{\begin{array}{c}1, if \quad r \le {R}_{1}\\ \frac{1}{2}\left[1+\mathrm{cos}\left(\pi \frac{r-{R}_{1}}{{R}_{2}-{R}_{1}}\right)\right]\\ 0, if \quad r \ge {R}_{2}\end{array}\right., if \quad {R}_{1}<r<{R}_{2},$$

5 $${b}_{ij}={\left(1+{\xi }_{ij}^{n}\right)}^{-\frac{1}{2n}},$$

6 $${\xi }_{ij}=\sum_{k\ne i,j}{f}_{C}({r}_{ij}){g}_{\text{mod}}\left[{\theta }_{ijk}\right]{\text{exp}}\left[\alpha {\left({r}_{ij}-{r}_{ik}\right)}^{m}\right],$$

7 $${g}_{\text{mod}}\left(\theta \right)={c}_{1}+{g}_{o}\left(\theta \right){g}_{a}\left(\theta \right),$$

8 $${g}_{o}\left(\theta \right)=\frac{{c}_{2}{\left(h-\mathrm{cos} \theta \right)}^{2}}{{c}_{3}+{\left(h-\mathrm{cos} \theta \right)}^{2}},$$

9 $${g}_{a}\left(\theta \right)={1+c}_{4}{\text{exp}}\left[-{c}_{5}{\left(h-\mathrm{cos} \theta \right)}^{2}\right],$$

where *E* is decomposed into bond energies *V*_*ij*_ between atoms *i* and *j*. *r*_*ij*_ is the distance between atoms *i* and *j*, *θ*_*ijk*_ is the angle confined by the bonds between *ij* and *ik*. The function *A*exp(-*λ*_1_*r*_ij_) and *b*_*ij*_*B*exp(− *λ*^*2*^*r*_*ij*_) represent a repulsive and an attractive term, respectively. The extra term *f*_*C*_ is merely a smooth cutoff function, to limit the range of the potential. *b*_*ij*_ represents a measure of the bond order. The term 1 + *ξ*_*ij*_ corresponds to the coordination number of atom *i*, where *ij* bond and other bonds are counted as 1 and *ξ*_*ij*_ respectively. The term *g*_mod_(*θ*) is the modified angular-dependent term. The potential parameters, *A*, *B*, *λ*_1_, *λ*_2_, *n*, *α*, *m*, *c*_1_, *c*_2_, *c*_3_, *c*_4_, *c*_5_, *h*, *R*_1_, and *R*_2_, are given in Table 1. The simulation box was a cubic cell with a side length of 50.00 Å. In the MD simulation, 6256 Si atoms were placed in the box as an initial configuration with in the *NVT* ensemble. A time step of 1 fs was used in the Verlet algorithm. The atomic configuration was initialized at random and the system was equilibrated at 3000 K for 500,000 steps. Then it was cooled to 300 K during 5,000,000 steps and annealed at 300 K for 500,000 steps. After the MD simulation, the configuration obtained was refined by RMC simulation with constraints on the coordination number and the Si–Si-Si bond angle distribution, in order to avoid formation of unfavorable disordered structures. The model reproduces the experimental X-ray *S*(*Q*).

**Table 1:** Potential parameters of the modified Tersoff potential.

*A* (J)	5.2577 × 10^−16^
*B* (J)	1.9386 × 10^−17^
*λ*_1_ (1/Å)	3.2300135
*λ*_2_ (1/Å)	1.3457970
*n*	1
*α*	2.3890327
*m*	1
*c*_1_	0.20173476
*c*_2_	730,418.72
*c*_3_	1,000,000
*c*_4_	1
*c*_5_	26

For *l*-Si, additional principles (FP)MD simulations within the framework of density functional theory were performed for a 64-atom Si supercell with periodic boundary conditions. The calculations were performed using the projector-augmented wave method^38^ and the generalized-gradient approximation with the exchange correlation functional of Perdew, Burke, and Ernzerhof^39^. The electronic wave functions were expanded in a plane-wave basis with an energy cutoff of 400 eV at the Γ point in the Brillouin zone. Atomic configurations selected from the FPMD trajectory generated using the Vienna Ab initio Simulation Package^40^ were used to validate the atomistic model of *l*-Si.

### Ring size distribution analysis

The ring size distribution was calculated using the R.I.N.G.S. code^41,42^ on the basis of the King^43^ and primitive^44,45^ criteria. The first coordination distances were set to 2.5 Å and 3.0 Å for *a*-Si and *l*-Si, and to 1.9 Å and 2.4 Å for *a*-SiO_2_ and *l*-SiO_2_, respectively.

### Cavity volume calculation

Cavity volume analyses were performed using the pyMolDyn code^46^. The code can calculate three different types of cavity, domain, center-based (Voronoi), and surface-based cavities. We calculated surface cavity volumes with a cut off distance *r*_c_ of 2.5 Å.

### Tetrahedral order parameter *q*

The tetrahedral order parameter for the Si-centered hyper-tetrahedra and SiO_4_ tetrahedra are defined as^47^

10 $$q\equiv 1-\frac{3}{8}\sum_{i=1}^{3}\sum_{k=i+1}^{4}{\left(\frac{1}{3}+{\mathrm{cos}} {\theta }_{ijk}\right)}^{2},$$

where *θ*_*ijk*_ is the angle formed between the central Si atom *j* and its neighboring Si or O atoms *i* and *k*. This parameter was designed to give a value of unity for a regular tetrahedron and a mean value of zero for a perfect gas.

## Results and discussion

**Figure 2:**
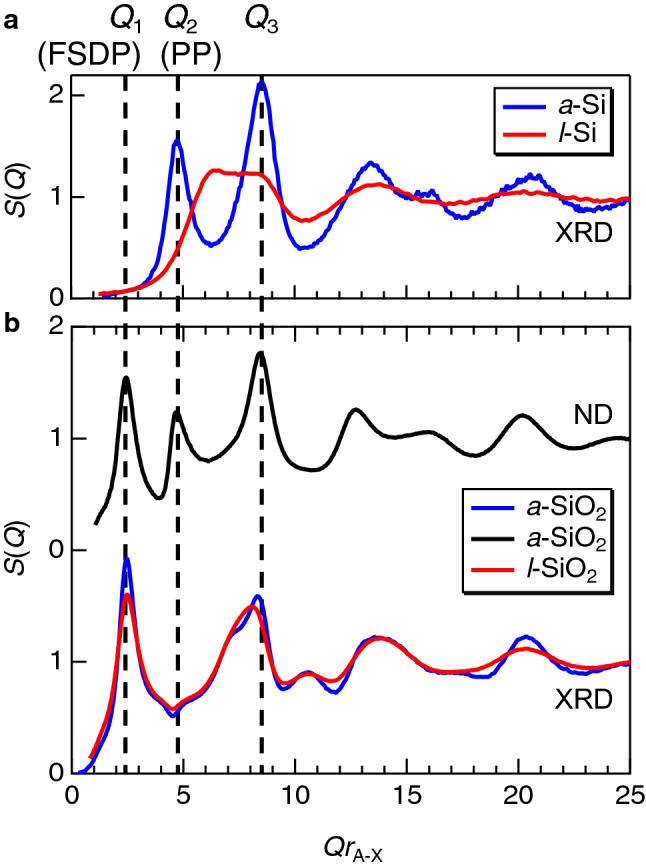
(**a**) X-ray structure factors, *S*(*Q*), for *a*-Si^8^ and *l*-Si (1770 K)^29.^. **(b)** X-ray structure factors, *S*(*Q*), f or *a*-SiO_2_^29^ and *l*-SiO_2_ (2019 K)^51^, together with neutron *S*(*Q*)^30^ for *a*-SiO_2_. Scattering vector *Q* is scaled by multiplying by *Qr*_A-X_ (first coordination distance) obtained by a Fourier transform of *S*(*Q*). *r*_A-X_ = *r*_Si-Si_ = 2.35 Å and 2.45 Å for *a*-Si and *l*-Si, and *r*_A-X_ = *r*_Si-O_ = 1.61 Å and 1.62 Å for *a*-SiO_2_ and *l*-SiO_2_, respectively.

The X-ray structure factor, *S*(*Q*), for *a*-Si^8^ is shown in Fig. 2a together with that of *l*-Si (1770 K)^29^. We observe *Q*_2_ (PP) and *Q*_3_ at *Qr*_A-X_ ~ 5 and 8.5, respectively, and no *Q*_1_(FSDP) is observed. Note that scattering vector *Q* is scaled by multiplying by *r*_A-X_ (first coordination distance)^6,11–15,18,22,24,29,48^ obtained by a Fourier transform of *S*(*Q*). *S*(*Q*) for *l*-Si is different from that for *a*-Si owing to the increased coordination number, *N*_Si-Si_, from approximately 3.9 (amorphous)^8^ to 5.7 (liquid)^29^ associated with the significant density increase from 2.30 g cm^−3^ (amorphous) to 2.57 g cm^−3^ (liquid). This behavior was also confirmed in previous FPMD studies^49,50^ and is consistent with the fact that *a*-Si is a semiconductor and *l*-Si is a metallic liquid. X-ray^29,30^ and neutron^30^ structure factors, *S*(*Q*), for *a*-SiO_2_ are shown in Fig. 2b. As mentioned in the previous section, *Q*_1_ (FSDP) is observed for *a*-SiO_2_ in both the X-ray and neutron *S*(*Q*), but *Q*_2_ (PP) is visible only in the neutron *S*(*Q*), because it reflects the packing fraction of oxygen atoms^12,15^ since neutrons are sensitive to O–O correlations but X-rays are more sensitive to Si–Si correlations. It is worth mentioning that the X-ray *S*(*Q*) of *l*-SiO_2_ (2019 K) is identical to that of *g*-SiO_2_. *Q*_1_ (FSDP) in the X-ray *S*(*Q*) shown in Fig. 2b is prominent in *l*-SiO_2_^51^, suggesting that Si–O covalent bonds are strong even in the liquid (2019 K). This behavior is consistent with the small differences in density (amorphous: 2.21 g cm^-3^, liquid: 2.10 g cm^-3^) and Si–O coordination number, *N*_Si-O_ (amorphous: 4.0, liquid: 3.9) between them. Considering the melting point of Si (1687 K)^52^ and SiO_2_ (1996 K)^53^, we have noted that the *S*(*Q*) of *l*-Si markedly changes at only 100 K above the melting point, although the temperature-dependent change in the diffraction pattern is small in *l*-Si^32^ (see Fig. S1). The marked change in structure above the melting point suggests a large difference in configuration entropy, which is evidence of the poor amorphous-forming ability. On the contrary, the *S*(*Q*) of *l*-SiO_2_ is similar to that of *g*-SiO_2_^51^. We assume that the change in the *S*(*Q*) is also an indicator of the amorphous-forming ability.

**Figure 3:**
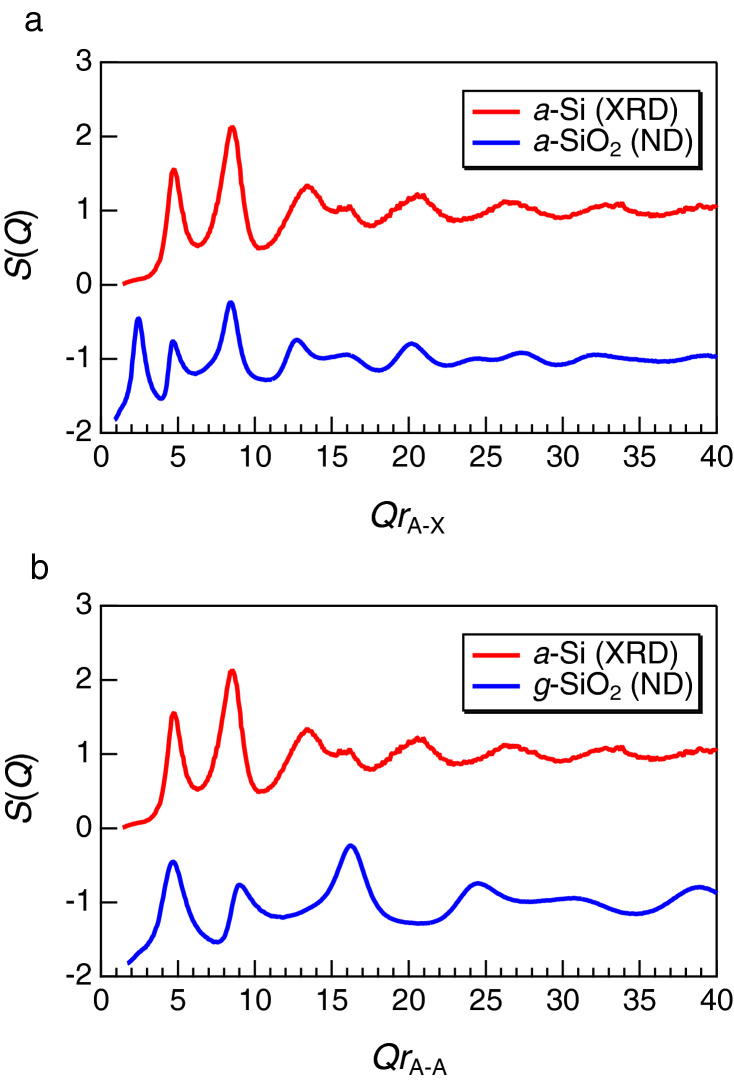
Structure factors *S*(*Q*) for *a*-Si and *a*-SiO_2_ scaled by **a** *r*_A-X_ (**a**) and (**b**) *r*_A-A_^29^. *r*_A-X_ for *a*-SiO_2_ is 1.61 Å, and *r*_A-A_ for *a*-SiO_2_ and *a*-Si are 3.10 Å and 2.35 Å, respectively.

Recently, Shi and Tanaka have reported a comparison of *S*(*Q*) of several disordered materials with a tetrahedral motif^25^, by scaling *r*_A-X_ and *r*_A-A_ (cation–cation distance). We also display the *S*(*Q*) of Si and SiO_2_ in Fig. 3. As can be seen in the figures, *a*-Si exhibits an FSDP when the *S*(*Q*) is scaled by *r*_A-A_, but not when scaled by *r*_A-X_. The scaling of *Q* is still an open question, but, recently Salmon and Zeidler have reported that *a*-Si does not have an FSDP^12^.

**Figure 4:**
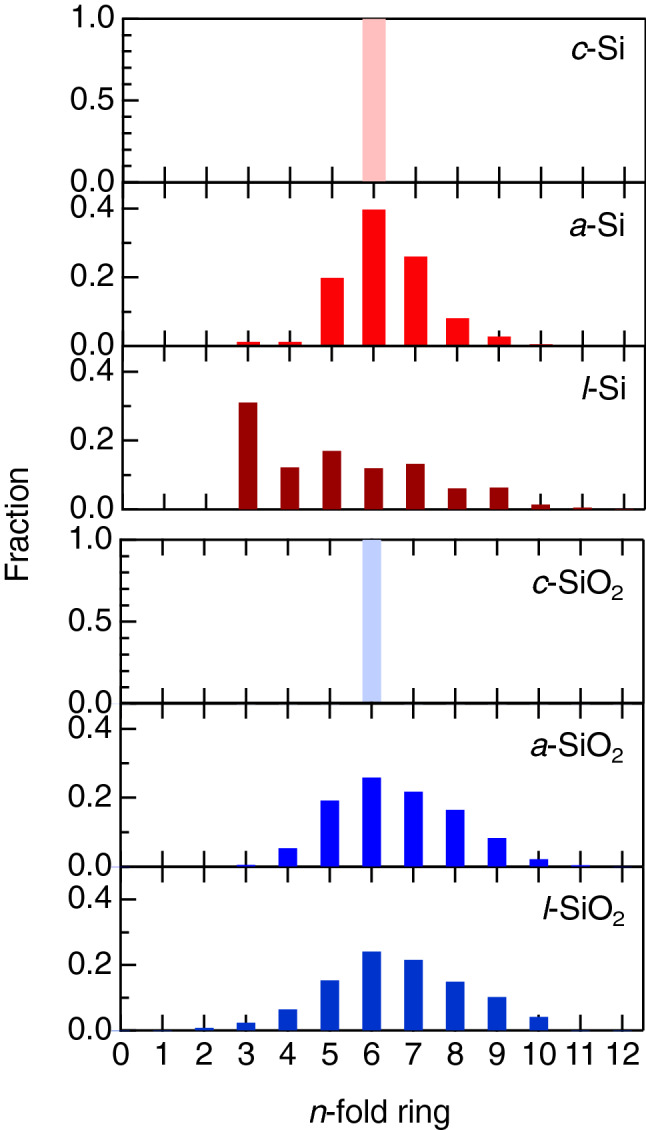
Primitive ring size distributions in a series of Si and SiO_2_. The results of *a*-Si, *l*-Si (1770 K), and *l*-SiO_2_ (2373 K) are calculated using a structural model obtained by combined molecular dynamics (MD)–reverse Monte Carlo (RMC) modeling^29^, RMC modeling^29^, and MD simulation^31^, respectively. The results of *a*-SiO_2_ are taken from ref. 29. It is confirmed that the ring size distribution of *l*-Si obtained by RMC modeling is identical to the result obtained by density functional (DF)–MD simulation shown in Fig. S3. Since the Si–O-Si bond angle is nearly straight^2^ in SiO_2_, we define that a sixfold ring consists of six SiO_4_ tetrahera, which allows us to decorate oxygen atoms in the analysis. Note that it is not appropriate to compare the ring statistics of *l-*Si with those of *a*-Si and SiO_2_, since the structure of *l*-Si is beyond the scope of the corner-sharing tetrahedra motif owing to a significantly increased Si–Si coordination number.

To understand the relationship between the diffraction peaks and the topology, we calculated ring size distributions on the basis of King and primitive criteria. The primitive and King ring size distributions for crystalline (*c*)-Si and SiO_2_ (cristobalite) calculated from crystal structures, and for structure models for liquid and amorphous Si and SiO_2_ obtained by computer simulations, are shown in Fig. 4 and Fig. S2, respectively. Note that the ring criterion does not affect the overall shape of the distributions. The crystalline phases of both Si and SiO_2_ exhibit only sixfold rings, in which the ring comprises six silicon atoms in *c*-Si, whereas it comprises six silicon atoms and six oxygen atoms (AXAX rings) in *c*-SiO_2_. The ring size distributions of *a*-Si and *a*-SiO_2_ are broad, and the sixfold rings are dominant for both *a*-Si and *a*-SiO_2_. The ring size distributions of *a*-Si are consistent with the result of a previous study reported by Opletal et al.^54^. It is stressed that the ring size distributions of *l*-Si are significantly different from those of *a*-Si. The large fraction of threefold rings in the liquid is due to the increased coordination number associated with a significant increase in the density of the liquid. This behavior of the ring size distribution is in line with the difference in diffraction data shown in Fig. 2. On the other hand, the ring size distributions of *l*-SiO_2_ (2373 K) are identical to those of *a*-SiO_2_, which is consistent with the similarity of the density and coordination number between them. The broad ring size distributions of *a*-SiO_2_ and *l*-SiO_2_ are a signature of topological disorder according to Gupta and Cooper^55^, and we previously hypothesized that the width of the ring size distribution is an indicator of the amorphous-forming ability^28,56^ when short-range structures are the same. Indeed, it appears that *a*-Si exhibits a narrower ring size distribution than *a*-SiO_2_, demonstrating that topological order/disorder is a suitable descriptor for understanding the amorphous-forming ability.

**Figure 5:**
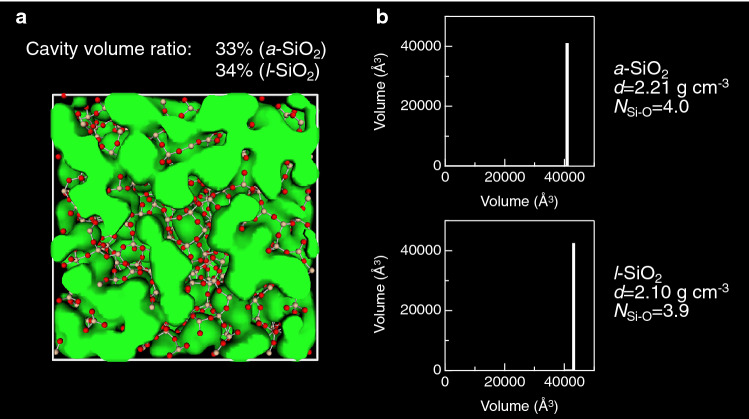
(**a**) Visualization of cavities in *a*-SiO_2_^29^ and (**b**) Cavity size distributions in *a*- and *l*-SiO_2_ (2373 K). The cavity volume ratio of *l*-SiO_2_ was calculated on the basis of the atomic configuration obtained by MD simulation. Only the *a*-SiO_2_ configuration is shown, because it is difficult to distinguish between *a*-SiO_2_ and *l*-SiO_2_ by visual inspection.

**Figure 6:**
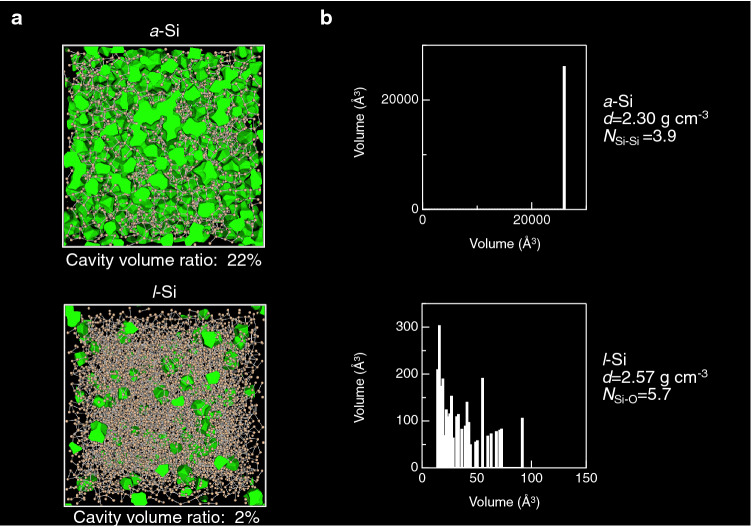
(**a**) Visualization of cavities and (**b**) cavity size distributions in *a*-Si and *l*-Si (1770 K).

The formation of rings is an important structural feature in covalent amorphous materials; hence, it results in the generation of cavities (empty spaces). We visualize a cavity (highlighted in green) of *a*-SiO_2_ together with the cavity size distributions in *a*-SiO_2_ and *l*-SiO_2_ (2373 K) in Fig. 5. The cavity volume ratio of *a*-SiO_2_ is approximately 33%^29^. The large cavity volume ratio is presumed to be a signature of covalent amorphous materials. Note that the cavity volume ratio of *l*-SiO_2_ is comparable to that of *a*-SiO_2_ owing to the small difference in density between them. The cavity size distributions in SiO_2_ indicate that both *a*- and *l*-SiO_2_ have a large cavity in disordered structure. Figure 6 visualizes cavities in *a-*Si and *l*-Si together with the cavity size distributions. The cavity volume ratio of 22% for *a*-Si is much smaller than that of 33% for *a*-SiO_2_ shown in Fig. 5, but we observe a significant reduction in cavity volume ratio in *l*-Si (from 22% in *a*-Si to 2% in *l*-Si), which is associated with the increase in density (2.30 to 2.57 g cm^−3^) and coordination number *N*_Si-Si_ (3.9 to 5.7). It is worth mentioning that the cavity size distributions indicated that a large cavity in *a*-Si is squeezed and only small cavities are observed in the liquid. This behavior suggests a poor amorphous-forming ability of Si, which is in line with the fact that *a*-Si can only be formed in a thin film.

**Figure 7:**
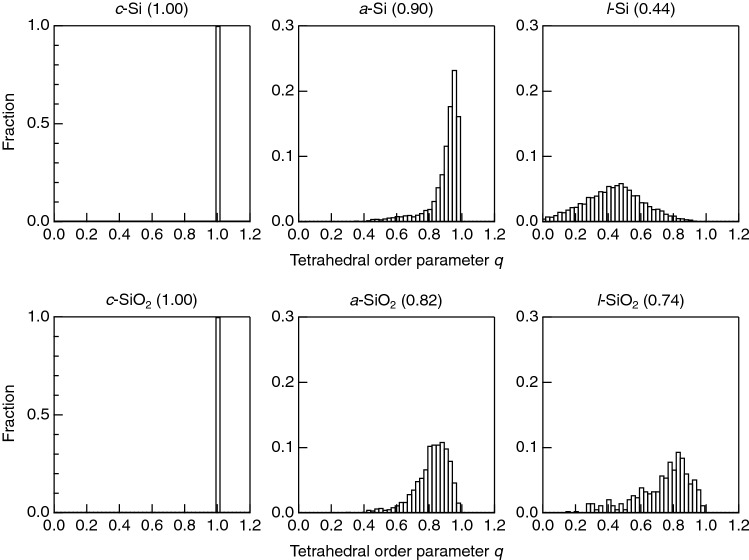
SiSi_4_ tetrahedral order parameter *q* for a series of silicon and silica. Average *q* values are given in round brackets. The SiSi_4_ *q* parameter of *a*-SiO_2_ remains almost the same in densified glass^2^. The SiO_4_ *q* parameters for *c*-, *a*-, and *l*-SiO_2_ are shown in Fig. S4. It is confirmed that the symmetry of SiO_4_ in *a*-SiO_2_ is better than that in *a*-Si, and SiO_4_ in *l*-SiO_2_ is more distorted.

Shi and Tanaka have recently focused on the symmetry of SiSi_4_ tetrahedra in *a*-Si and SiSi_4_ hyper-tetr ahedra in *a*-SiO_2_^25^. We apply this analysis to crystalline and amorphous Si and SiO_2_. Figure 7 shows *q* values of a series of Si and SiO_2_. Both *c*-Si and *c*-SiO_2_ have perfect tetrahedra as indicated by the average *q* value of 1. A comparison between *a*-Si and *a*-SiO_2_ demonstrates that SiSi_4_ hyper-tetrahedra in *a*-SiO_2_ are highly distorted in comparison with regular SiSi_4_ tetrahedra in *a*-Si, as revealed by the average *q* values of *a*-SiO_2_ being much smaller than that for *a*-Si of 0.90. In addition, the distribution of *q* values is broader in *a*-SiO_2_. It is also suggested that hyper-tetrahedra in *l*-SiO_2_ are slightly distorted in comparison with those in *a*-SiO_2_, whereas the average *q* value of *l*-Si is the smallest owing to the formation of fivefold and sixfold Si in the liquid. The profile of the *q*-parameter for *l*-Si is consistent with the profile reported in a previous FPMD study^49,50^. Therefore, the structural motif in the liquid phase is completely different between Si and SiO_2_ as indicated by the behavior of the density, since the density difference between *a*-SiO_2_ and *l*-SiO_2_ is small, but large between *a*-Si and *l*-Si.

Thus, we have revealed the differences in terms of diffraction peaks and the topology by a combination of diffraction measurements and topological analyses. We show that the diffraction data and the topology for *a*-Si and *l*-Si are very different. This result is in sharp contrast to SiO_2_, in which both the diffraction pattern and topology are identical for *a*-SiO_2_ and *l*-SiO_2_. We are confident that this behavior is the reason why SiO_2_ is a glass former and Si is not. In other words, the tetrahedral corner-sharing network of AX_2,_ in which A is a fourfold cation and X is a twofold anion, as indicated by the FSDP, is an important motif for the amorphous-forming ability that can rule out *a*-Si as a good amorphous former. This concept is consistent with the fact that it is impossible to form an elemental bulk amorphous material using melt quenching technique^57^.

As mentioned in the introduction, the FSDP and PP are suitable descriptors of an amorphous network. The typical behavior of the FSDP and PP under high pressure shown in Fig. 1, in which the FSDP diminishes with a peak shift to a higher *Q* and the PP becomes sharp with increasing pressure at room temperature, without any peak shifts (Fig. 1a). Onodera et al*.* have recently reported the evolution of the FSDP with a peak shift in *a*-SiO_2_ owing to an LDA-HDA transition induced by heating under high pressure (see Fig. 1b)^2^. No such significant peak shift can be observed in *a*-Si under pressures owing to the very strong fully tetrahedral covalent network associated with the absence of an FSDP (note, however, that a first-order-like transformation to high-density *a*-Si occurs above 10 GPa^58–61^). Therefore, it is suggested that the first peak in the *S*(*Q*) of *a*-Si can be assigne d to a PP, because the peak does not show any shifts under high pressures. This interpretation supports that FSDP is a signature of amorphous formin g ability in amorphous network system, although FSDP is not a signature of network^29^. On the other hand, the FSDP of liquid phosphorus diminishes under high pressures and temperatures, which is associated with the network formation of P_4_ molecules by transition^62,63^.

In this article, we demonstrate that topological analyses are powerful tools for understanding the diffraction peaks of disordered materials. Our conclusion fully supports the discussion of Price et al. in 1988^13^. Understanding the diffraction peaks of disordered materials via a series of topological analyses will give rise to the capability to forge a new path for designing novel functional disordered materials.

## Supplementary Information

Supplementary Information.
