# Supplementary material for: Relationship between diffraction peak, network topology, and amorphous-forming ability in silicon and silica
Source: Sci Rep. 2021 Nov 12;11:22180. doi: 10.1038/s41598-021-00965-5 (PMC8590056; doi:10.1038/s41598-021-00965-5)
Supplement: Supplementary file 1 — Supplementary Information. [file 41598_2021_965_MOESM1_ESM.pdf]

**Supplemental Information for**

# Relationship between diffraction peak, network topology, and amorphous-forming ability in silicon and silica

by

S. Kohara *et al.*

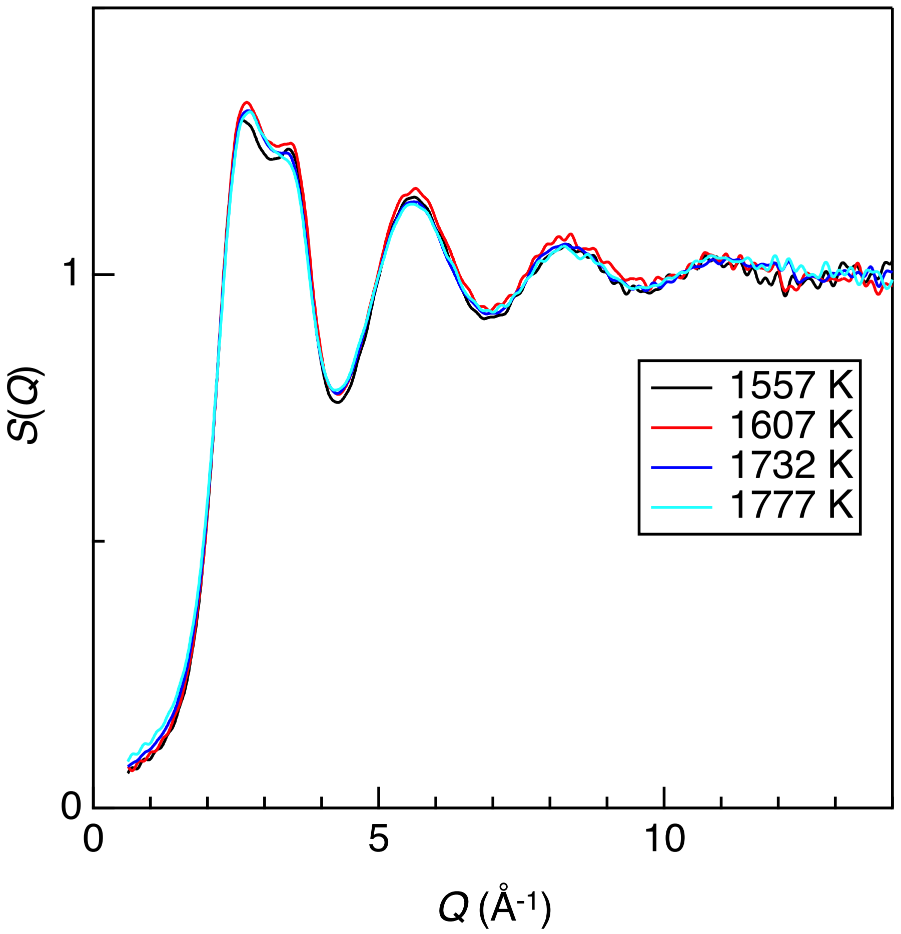


**Figure S1** X-ray $S(Q)$ of *l*-Si measured at 1557 K, 1607 K, 1732 K, and 1777 K.

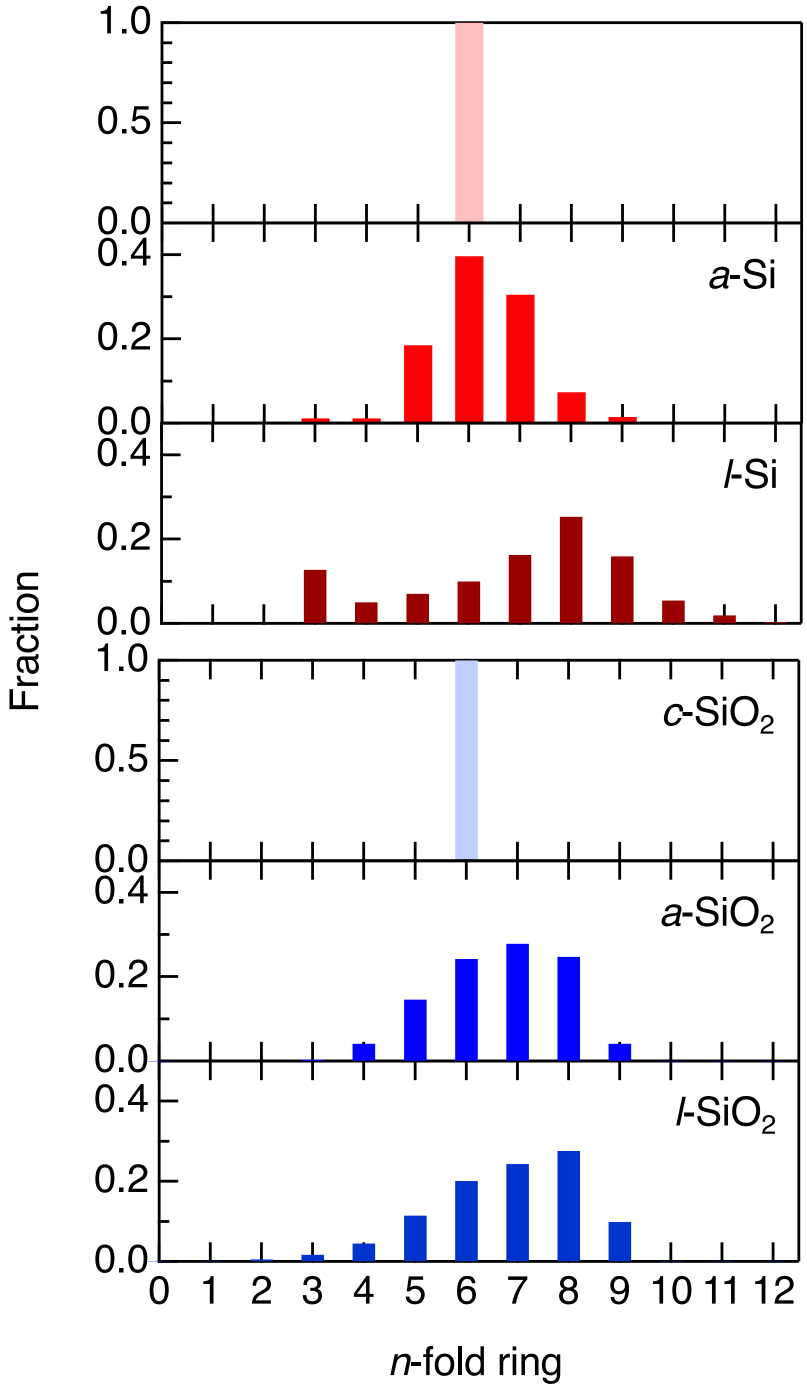


**Figure S2** King ring size distributions in a series of Si and $SiO_2$.

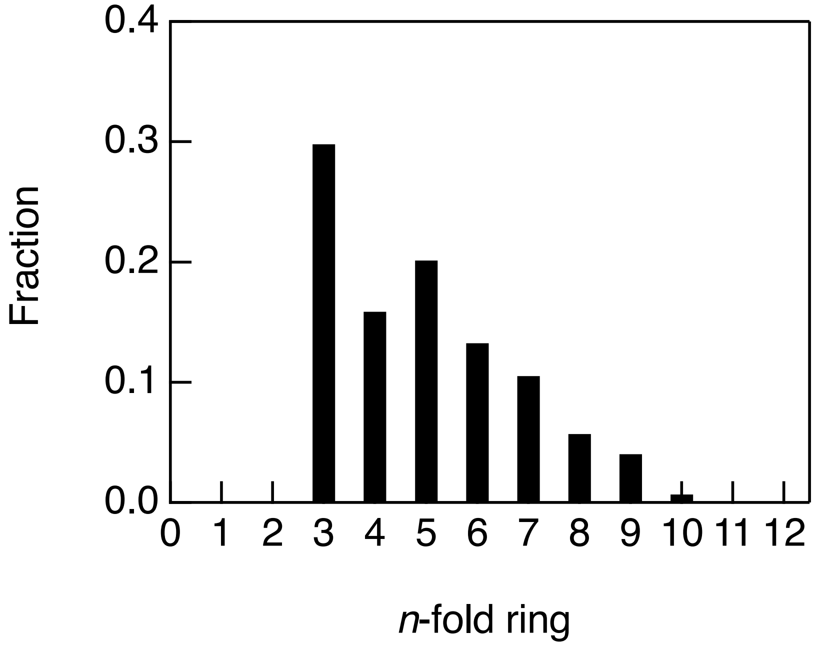


**Figure S3** Primitive ring size distributions for *l*-Si (1770 K). The atomic configuration of the liquid was obtained by DF–MD simulation.

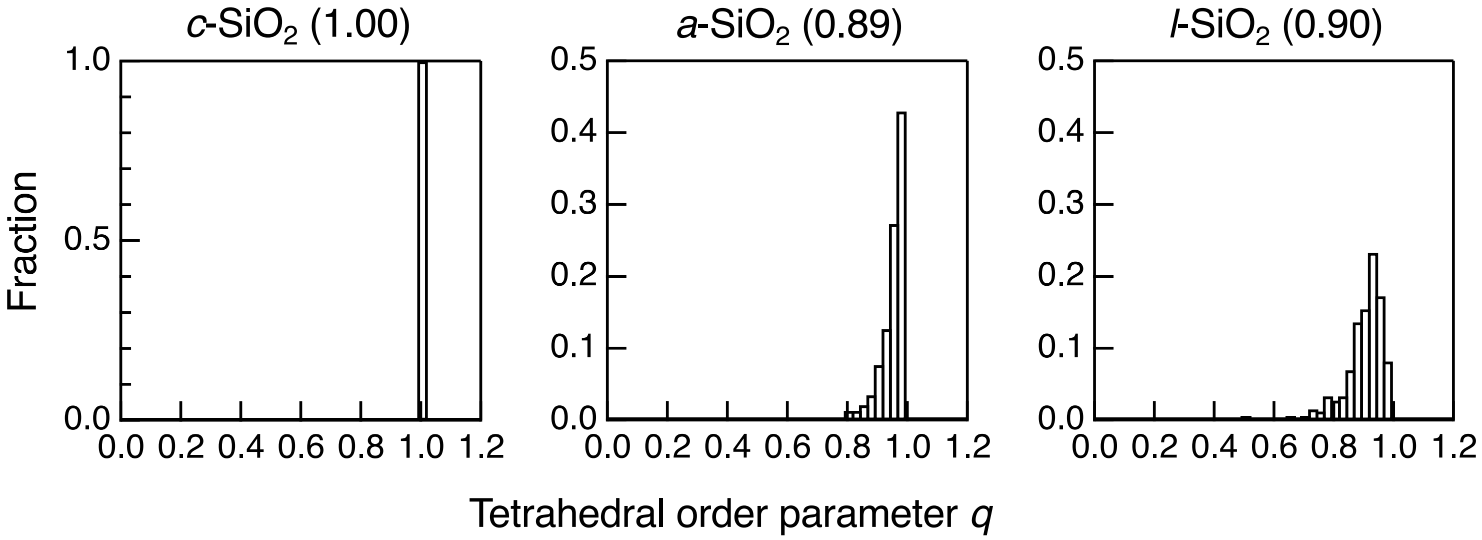


**Figure S4** $SiO_4$ tetrahedral order parameter *q* for a series of Si and $SiO_2$. Average *q* values are given within parentheses.
